# Identification of the gene for β-fructofuranosidase from *Ceratocystis moniliformis* CMW 10134 and characterization of the enzyme expressed in *Saccharomyces cerevisiae*

**DOI:** 10.1186/1472-6750-13-100

**Published:** 2013-11-14

**Authors:** Niël van Wyk, Kim M Trollope, Emma T Steenkamp, Brenda D Wingfield, Heinrich Volschenk

**Affiliations:** 1 Department of Microbiology, Stellenbosch University, Room A322, JC Smuts Building, De Beer Street, Private Bag X1, Matieland 7602 Stellenbosch, South Africa; 2 Department of Microbiology and Plant Pathology, Forestry and Agricultural Biotechnology Institute, University of Pretoria, Pretoria, South Africa

**Keywords:** β-fructofuranosidase, Short-chain fructooligosaccharides, *Ceratocystis moniliformis*, *Saccharomyces cerevisiae*, Heterologous expression

## Abstract

**Background:**

β-Fructofuranosidases (or invertases) catalyse the commercially-important biotransformation of sucrose into short-chain fructooligosaccharides with wide-scale application as a prebiotic in the functional foods and pharmaceutical industries.

**Results:**

We identified a β-fructofuranosidase gene (*CmINV*) from a *Ceratocystis moniliformis* genome sequence using protein homology and phylogenetic analysis. The predicted 615 amino acid protein, CmINV, grouped with an existing clade within the glycoside hydrolase (GH) family 32 and showed typical conserved motifs of this enzyme family. Heterologous expression of the *CmINV* gene in *Saccharomyces cerevisiae* BY4742∆*suc2* provided further evidence that CmINV indeed functions as a β-fructofuranosidase. Firstly, expression of the *CmINV* gene complemented the inability of the ∆*suc2* deletion mutant strain of *S. cerevisiae* to grow on sucrose as sole carbohydrate source. Secondly, the recombinant protein was capable of producing short-chain fructooligosaccharides (scFOS) when incubated in the presence of 10% sucrose. Purified deglycosylated CmINV protein showed a molecular weight of ca. 66 kDa and a *K*_m_ and *V*_max_ on sucrose of 7.50 mM and 986 μmol/min/mg protein, respectively. Its optimal pH and temperature conditions were determined to be 6.0 and 62.5°C, respectively. The addition of 50 mM LiCl led to a 186% increase in CmINV activity. Another striking feature was the relatively high volumetric production of this protein in *S. cerevisiae* as one mL of supernatant was calculated to contain 197 ± 6 International Units of enzyme.

**Conclusion:**

The properties of the CmINV enzyme make it an attractive alternative to other invertases being used in industry.

## Background

Invertases (β-d-fructofuranosidases, EC 3.2.1.26), widely distributed among plants and microorganisms, catalyse the hydrolysis of sucrose to equimolar amounts of d-fructose and d-glucose [1]. At saturated (high) sucrose concentrations, many β-fructofuranosidases – especially those of fungal origin – display varying degrees of fructosyltransferase activity by cleaving the β-(2 → 1) linkage, releasing glucose, and transferring the fructose moiety onto an acceptor molecule other than water. These acceptor molecules are initially usually unhydrolysed sucrose (GF) which generates 1-kestose (GF2), whereafter another fructose moiety can be transferred onto 1-kestose to produce 1-nystose (GF3). This fructosyltransferase reaction can continue onto 1-nystose to produce 1^F^-fructofuranosylnystose (GF4) [2].

Collectively known as short-chain fructooligosaccharides (scFOS), mixtures of GF2-GF4 are considered as health-promoting food ingredients [3]. ScFOS have a lower sweetness intensity than sucrose making it suitable as a diabetic-friendly sweetener with low caloric and non-cariogenic properties. More importantly, scFOS function as prebiotic compounds that selectively stimulate beneficial colonic bifidobacteria and lactobacilli [4]. As a dietary additive they exert an array of beneficial effects on human health ranging from protection against the development of colon cancer and inflammatory bowel disease, improved calcium absorption, reduction of serum lipids and diminishing adverse effects of intestinal pathogens [3].

Furthermore, β-fructofuranosidases are currently also being investigated as potential biosensors for the detection of various biologically and medically relevant compounds [5,6]. In general, this application relies on the target-induced release of a β-fructofuranosidase moiety from a functional DNA-enzyme conjugate. β-Fructofuranosidases are preferred above other types of enzymes due to the relatively inexpensive substrate, easily detectable end-products and high catalytic activities of many fungal β-fructofuranosidases. For this reason it is desirable to produce high levels of β-fructofuranosidases in pure form.

β-Fructofuranosidases belong to the glycoside hydrolase family 32 (GH32) of the sequence-based classification of carbohydrate-active enzymes [7]. This family of enzymes also includes enzymes that exhibit activity on fructan-based carbohydrates like inulinases (EC 3.2.1.7), levanase (EC 3.2.1.65), exo-inulinase (EC 3.2.1.80), transfructosidases like sucrose:sucrose 1-fructosyltransferase (EC 2.4.1.99) and fructan:fructan 1-fructosyltransferase (EC 2.4.1.100). Members of the GH32 enzyme family retain the anomeric configuration of the anomeric carbon by operating through a double displacement mechanism. In all β-fructofuranosidases, the catalytic amino acid residues are conserved in that an aspartic acid (D) residue conducts the nucleophilic attack and a glutamic acid (E) residue acts as the general catalytic acid/base. These two bases are often located within conserved regions known as the WMN**D**PNG and **E**C motifs, respectively. Another aspartic acid residue within a conserved R**D**P motif has been identified as the transition state stabilizer and forms part of what is known as the catalytic triad with the other two residues [8].

Based on the 3D-stuctures that have been resolved for GH32 enzymes, the N-terminal catalytic domains exhibit a β-propeller configuration, a feature shared with family GH68 (which contains bacterial fructan-active enzymes) and together these two families form the GH-J clan [9]. The GH32 enzymes that have been described have bimodular arrangements and usually also have a C-terminal β-sandwich domain, which seems to maintain structural stability and may have a role in protein oligomerization [10].

A growing demand for scFOS as food and pharmaceutical ingredient combined with the inherent limitations of available enzymes used in the commercial production of scFOS from sucrose have been the driving force behind extensive research efforts to develop cost-effective production and application of β-fructofuranosidases [11]. Here we describe the molecular, phylogenetic, and biochemical characterization of the first extracellular β-fructofuranosidase, CmINV, from the fungus *Ceratocystis moniliformis* (strain CMW 10134) recombinantly produced at high levels in an invertase-negative strain of *S. cerevisiae*.

## Results

### Cloning of *CmINV* and *fopA* genes

The primers designed in this study correctly amplified the *CmINV* gene from genomic DNA of *C. moniliformis*. For heterologous expression of the *CmINV* gene, no cDNA isolation of the gene was required as the gene contained no predicted introns. Functionality of the *CmINV* gene was confirmed in *S. cerevisiae* BY4742 as the gene complemented the *suc2* gene knock-out that prohibits the strain from growing on sucrose as sole carbohydrate source. The synthetically-made *fopA* gene also complemented the *suc2* gene knock-out when expressed in *S. cerevisiae* BY4742, as reported previously [12].

*In silico* translation of the *CmINV* reading frame obtained revealed a corresponding 615 amino acid polypeptide with a calculated molecular mass of 65.7 kDa. The first 19 residues of the translated sequence comprised a eukaryotic secretion signal. In addition, the sequence of the mature protein had a predicted pI of 4.77 and seven putative *N*-glycosylation sites were also identified on the mature sequence.

### Alignment and phylogeny

**Figure 1:**
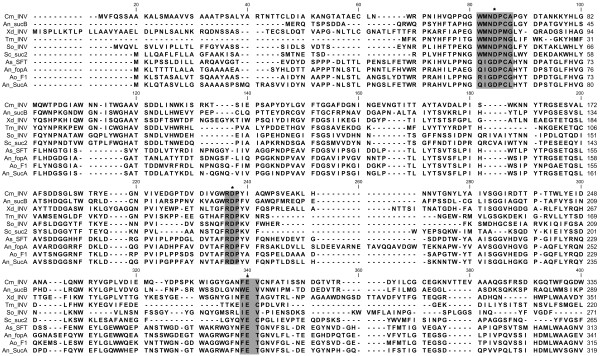
**Multiple sequence alignment of the N-terminal β-propeller-containing end from selected β-fructofuranosidases.** The GenBank accession number of the protein sequences are as follows: An_SucB ABB59679 from *A. niger*; Xd_INV ACL79833 from *Xanthophyllomyces dendrorhous*; Tm_INV CAA04518 from *Thermotoga maritima*; So_INV ADN34605 from *Schwanniomyces occidentalis*; Sc_suc2 NP_012104 from *S. cerevisiae*; As_SFT CAB89083 from *Aspergillus sydowii*; An_fopA BAB67771 from *A. niger*; Ao_F1 ABW87267 from *A. oryzae*; An_SucA ABB59678 from *A. niger* (*A. japonicus*). The catalytic triad of the sequences are framed with the asterisks (*) indicating the acidic amino acids acting as catalytic residues. Alignment of sequences was conducted on CLC Genomics Workbench 6.0.5 (CLC Bio).

**Figure 2:**
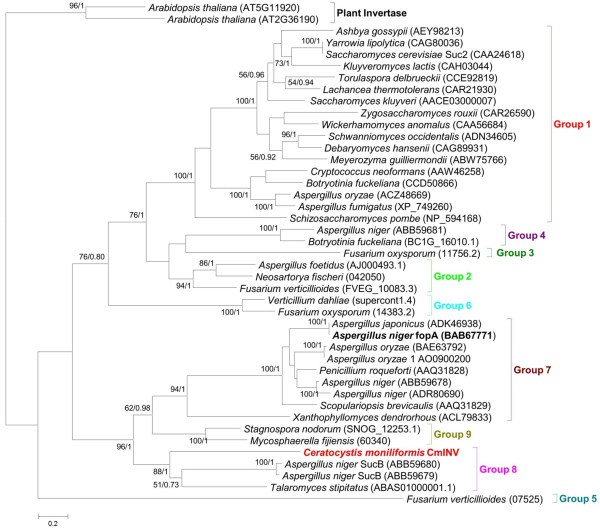
**Maximum likelihood phylogeny of GH32 protein family representatives.** The *Ceratocystis moniliformis* protein, CmINV, is indicated bold red and the *Aspergillus niger* protein, fopA, is indicated in bold black. The group designations (Group 1-9) are indicated with square brackets and are based on those proposed by Parrent et al. [15]. Bootstrap support values based on 1000 replicates and Bayesian posterior probabilities are respectively indicated above or below individual branches.

Alignment of the CmINV amino acid sequence with known invertases revealed that the *CmINV* gene encodes a product with the expected conserved motifs reported for other invertases and members of the glycoside hydrolase family 32 (Figure 1). Based on these alignments, the residues D66, D205 and E283 of the translated protein sequence, were identified as the nucleophile, transition-state stabilizer and general acid/base catalyst, respectively. The WMN**D**PNG conserved region which includes the nucleophilic residue differs slightly in that the last two residues are CA instead of NG. However, the CmINV sequence has more homology to this region than other β-fructofuranosidases with known strong fructosyltransferase activities like fopA, Suc1 from *A. niger*[13] and F1 from *Aspergillus oryzae*[14]. Within the existing phylogenetic framework for the GH32 family [15], the CmINV protein sequence was grouped in a well-supported clade which was previously designated as the Group 8 intracellular invertase subfamily (Figure 2). The closest related protein (with 28.4% amino acid identity) to CmINV that has been characterized previously is the intracellular β-fructofuranosidase, SucB, of *Aspergillus niger*[16].

### Purification of CmINV and kinetic parameters

Following size-exclusion chromatography of the crude yeast supernatant, electrophoretically homogenous β-fructofuranosidase-positive fractions were used for further characterization (see Additional file 1). Initial rate enzyme reactions of CmINV with increasing sucrose concentrations led to typical Michaelis-Menten-like kinetics with the apparent Michaelis constant (*K*_m_) and maximal reaction velocity (*V*_max_) parameters calculated to be 7.50 mM and 986 μmol/min/mg protein, respectively.

### Influence of temperature and pH on CmINV hydrolytic activity

**Figure 3:**
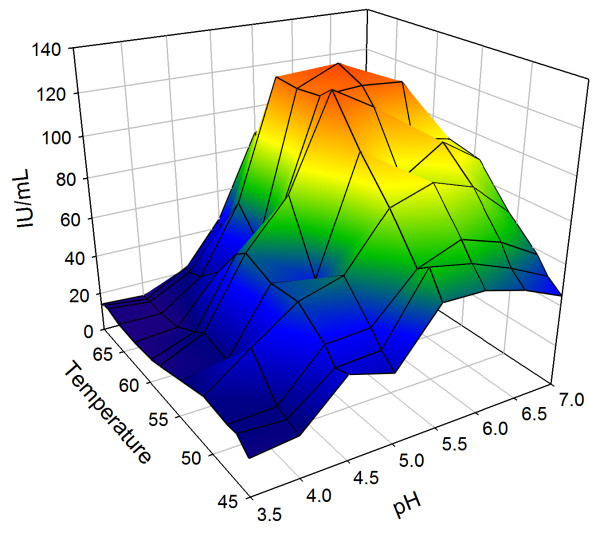
**3D-mesh plot representing pH and temperature optimum conditions of CmINV activity on sucrose.** Five minute assays were conducted using yeast culture supernatant incubated with 50 mM sucrose at temperatures ranging from 35-69°C and different pH values. Volumetric units IU/mL were defined as the amount of enzyme per millilitre producing 1 μmole of product per minute.

**Figure 4:**
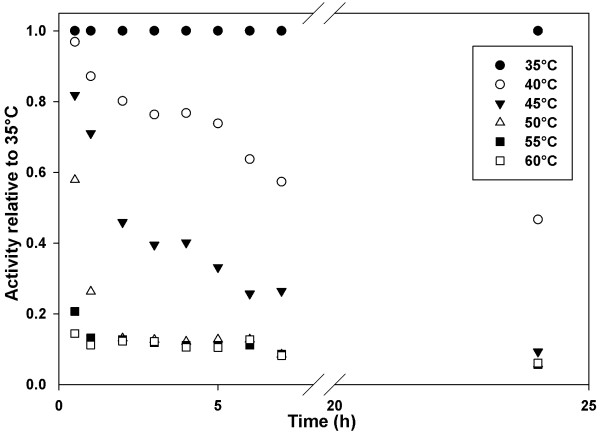
**Thermostability activity profile of purified CmINV.** Enzyme in 50 mM citrate-phosphate buffer (pH 6) was preincubated at abovementioned temperatures and five microliters were removed at each time point. A standard five minute assay was conducted on the preincubated enzyme at 60°C. Data points represent the average of quadruplicate assay values.

Purified CmINV was assayed for five minutes at various temperature and pH conditions simultaneously. The highest hydrolytic activity was recorded at a temperature of 62.5°C and a pH of 6.0 (Figure 3). Although CmINV hydrolytic activity could be detected at all the temperatures tested, no activity could be detected below pH 4.0. For thermostability analyses, the purified enzyme was preincubated at different temperatures for up to 24 hours before a five minute assay at 60°C was performed to generate a thermostability profile of the purified CmINV (Figure 4). CmINV did not retain any activity after 24 hours of incubation at temperatures over 40°C. Of note is that activity of the enzyme was already lost after 30 minutes of incubation at 60°C, which is just under its optimal temperature.

### Influence of ions on CmINV hydrolytic activity

The addition of Li^+^, Fe^3+^ and Co^2+^ ions at a concentration of 5 mM slightly increased CmINV hydrolytic activity (Table 1). However, a 186% increase in CmINV hydrolytic activity was observed in the presence of 50 mM Li^+^ ions. In all other cases, the treatments with ions led to a reduction in CmINV hydrolytic activity. The addition of 50 mM NH_4_^+^, Zn^+^ and Mg^2+^ completely inactivated the enzyme.

**Table 1:** Influence of several metal ions on purified CmINV activity ± SE values

**Ion present**	Percentage activity retained^*^	
	**5 mM**	**50 mM**
None	100%	100%
CaCl_2_	86 ± 6%	29 ± 2%
CoCl_2_	118 ± 1%	51 ± 1%
FeCl_3_	137 ± 7%	34 ± 2%
LiCl	119 ± 2%	186 ± 2%
MgCl_2_	94 ± 6%	5 ± 5%
NH_4_Cl	105 ± 16%	4 ± 0%
NiCl_2_	47 ± 2%	18 ± 2%
ZnCl_2_	80 ± 18%	4 ± 3%

^*^Standard five minute assays were conducted at 62.5°C and pH 6 using two different ion concentrations.

### ScFOS production

**Figure 5:**
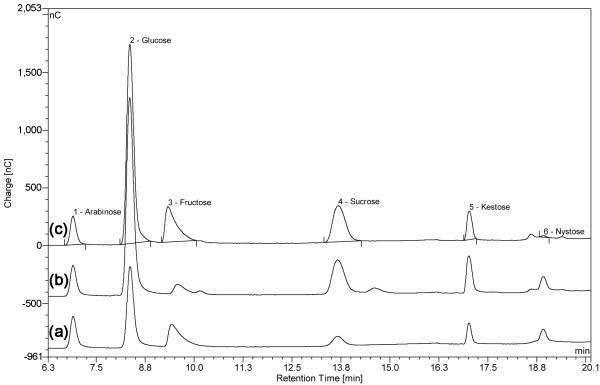
**HPAEC chromatograms of sugars released from sucrose by fopA and CmINV enzymes.** HPAEC chromatograms showing external standards **(a)** and products from the incubation of fopA **(b)** and CmINV **(c)** enzymes (1 IU) with 50 mM sucrose in 50 mM citrate phosphate buffer (pH 6) for 60 minutes. Samples **(b** and** c)** shown here were diluted 1000× from the original reaction.

Crude CmINV was capable of producing both 1-kestose and traces of 1-nystose from 10% sucrose under the experimental conditions (Figure 5). Its U_t_/U_h_ ratio i.e. fructosyltransferase to hydrolase activity was calculated to be 0.92. This was less than the U_t_/U_h_ ratio of 1.50 of fopA under the same conditions.

### Electrophoresis and zymogram analyses

**Figure 6:**
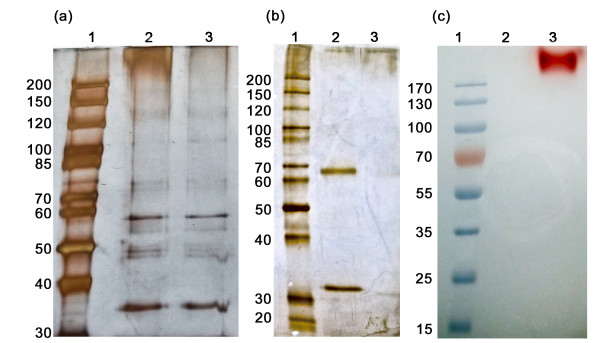
**SDS-PAGE and zymogram gels of CmINV. (a)** Silver-stained 10% polyacrylamide gel of crude extract of *S. cerevisiae* BY4742[CmINV] in lane 2. Lane 3 is a reference *S. cerevisiae* BY4742[pJC1] strain containing the empty vector pJC1. **(b)** Silver-stained 10% polyacrylamide gel of purified CmINV. Lane 2 contains the de-N-glycosylated version (lower band corresponds to PNGase F). Lane 3 contains the un-de-N-glycosylated version of purified CmINV. **(c)** Zymogram gel of purified CmINV. Lanes were loaded similar to gel **b**. The protein molecular weight markers PageRuler unstained Protein Ladder (lanes 1 in **a** and **b**) and PageRuler Prestained Protein Ladder (Thermo Scientific) (lane 1 in **c**) were used in SDS-PAGE and Zymogram analyses, respectively.

SDS-PAGE of *S. cerevisiae* BY4742[CmINV] supernatant showed a distinct band larger than 200 kDa (Figure 6). This band showed invertase activity when the same sample was subjected to zymogram analysis. De-*N*-glycosylation of the supernatant revealed a protein band (estimated at 66 kDa) corresponding to the predicted molecular size of the mature protein of 65.6 kDa. Despite several alterations to the protocol, the de-*N*-glycosylated version of CmINV showed no activity upon zymogram analysis.

### Small-scale recombinant production of CmINV and fopA enzymes

Supernatants of *S. cerevisiae* BY4742[CmINV] cultivated in 50 mL of SC media in 250 mL Erlenmeyer flasks for two days were calculated to produce 197 ± 6 IU of CmINV enzyme when assayed for five minutes at 60°C. In comparison, only 2.1 ± 0.5 IU of enzyme of *S. cerevisiae* BY4742[fopA] was produced under the same cultivation conditions by the yeast.

## Discussion

The bioconversion of sucrose by β-fructofuranosidases from *Aspergillus niger* or *Aureobasidium pullulans* is one of the current methods used in commercial scFOS production [17]. With the increasing demand for scFOS in food and pharmaceutical products, efforts to find alternative β-fructofuranosidases from other microbial sources or to increase enzyme production efficiency by known β-fructofuranosidase producers have been a major research thrust to lower scFOS production costs [18].

The recent availability of the complete genome sequence (unpublished) of *C. moniliformis* CMW 10134 facilitated the identification the *CmINV* gene, which encodes a β-fructofuranosidase. Phylogenetic analysis of this protein revealed that it forms part of a clade (Group 8) containing other known and putative β-fructofuranosidases [15]. Within Group 8, CmINV is closely related to the β-fructofuranosidase SucB of *A. niger*, although the members of Group 8 are thought to represent intracellular invertases because of their apparent lack of signal peptide cleavage sites [16]. Like the commercially-used proteins fopA from *A. niger* and Suc2 from *S. cerevisiae*, CmINV harbours a signal peptide cleavage site between amino acids 19 and 20, supporting the fact that it is a secreted enzyme. Experimental data substantiate this claim, as β-fructofuranosidase activity was detected in the supernatant of *S. cerevisiae*. However, Suc2 and fopA respectively form part of Groups 1 and 7 that both include other extracellular fungal invertases [14]. These data thus suggest that, in the case of the GH32 family, phylogenetic affinity alone has little predictive value regarding subcellular localization.

Since non-sucrolytic strains of *S. cerevisiae* have previously been shown to be ideal expression hosts for β-fructofuranosidase genes [12,16], *S. cerevisiae* BY4742∆*suc2* was used for the heterologous expression of the *CmINV* gene for ease of characterization of the protein. CmINV was purified from the supernatant of the recombinant yeast with size-exclusion chromatography. Since *S. cerevisiae* produces few extracellular proteins and the CmINV protein appeared electrophoretically homogenous on a silver-stained PAGE-gel, additional purification was not necessary for subsequent characterization of the protein. CmINV acts as a β-fructofuranosidase, which is able to hydrolyse the glucose-fructose glycosidic linkage in sucrose. CmINV appears to have a relatively high affinity for sucrose with an experimentally determined the *K*_m_ value of the purified CmINV for sucrose (7.50 mM) lower than what has been reported for the widely-used industrial invertase of *S. cerevisiae*, Suc2 [19-21]; Lafraya et al. [22] recently reported the *K*_m_ of 38 mM for Suc2, although varying *K*_m_ values for Suc2 ranging from 9.1 to 61.2 mM have been reported. The (*V*_max_) of CmINV for sucrose as substrate was 986 μmol/min/mg which is about 150 times higher than that for its closest characterized relative i.e. the SucB of *A. niger* with a *V*_max_ of 6.6 μmol/min/mg [16]. Calculation of the turnover rate (k_cat_) of the enzyme was not done due to the high level of recombinant glycosylation of CmINV, making accurate molecular weight determination misleading.

CmINV retained 75% of its hydrolytic activity observed under optimal pH and temperature conditions at pH levels of 5.5-6.5 and temperatures of 50-65°C. Although its closest characterized relative, the intracellular SucB of *A. niger*, possesses a similar pH optima profile, its temperature optima was only recorded at 37-40°C [16]. A characteristic that CmINV shares with Suc2 is its weak thermostability after prolonged exposure to higher-than-ambient temperatures [23]. A more thermostable version of an enzyme is highly desirable as it would increase the productivity of enzyme conversion [24]. One way to improve the thermostability of CmINV could be to add bifunctional cross-linkers as had been done to improve the thermostability of Suc2 [23]. Immobilizing invertases on several hydrophilic matrices have also been shown in many cases to improve the general stability of the enzyme [25].

This study showed that CmINV produce significant amounts of scFOS from sucrose. However, based on the experimental conditions, the fructosyltransferase to hydrolysis ratio was found not to be as high as the commercially-used fopA enzyme. Optimization of the experimental conditions could improve the fructosyltransferase reaction of the CmINV enzyme and subsequent scFOS yields. Since there is a noticeable difference in homology between the conserved region which contains the nucleophilic aspartic acid of CmINV and fopA, mutations within this region might be key if improvement of the fructosyltransferase capability is desired. This strategy was employed with *S. cerevisiae*’s Suc2 where most enhanced fructosyltransferase activity was observed when mutations were introduced within this conserved region [22].

Most of the metal ion additions to the enzyme reactions, with the exception of Li^+^, behaved predictably since most treatments had either a marginal improvement or negative impact [26,27]. To our knowledge, incorporating Li^+^ ions to any invertase reaction has not yet been reported and it is still unclear why the large increase in activity was observed. No particular pattern could be observed with regards to the ionic radius of Li^+^ relative to the other ions tested as the sizes of ionic radii of ions have been reported to have a varying effect on many enzymatic reactions [27]. Monovalent salts such as LiCl generally modifies the ionic strength of a solution which, depending on the charge distribution of the enzyme, have either a stabilizing or destabilizing impact on the enzyme conformation [28]. Although Ca^2+^, Mg^+^ and Na^+^ additions were shown to enhance invertase activity previously [26,29], the opposite was found for CmINV. Na^+^ additions were not conducted as the enzyme was already resuspended in Na^+^-containing PBS-buffer. Although ion additions clearly affect invertase activity, the results obtained from this study as well as from many previous reports indicate that no clear pattern exists to predict which ion will lead to a dramatic improvement or reduction in invertase activity.

Despite numerous theories, to predict at what levels a recombinant protein will be produced in its recombinant host is impossible [30,31]. This is mainly due to the complex interactions of all the factors that are involved in the successful production of any protein regardless whether it is foreign or not. Here, we show that the CmINV protein with its unmodified gene sequence can be produced at relatively high levels in *S. cerevisiae*. This is exemplified when compared to the fopA enzyme levels – functionally similar and expressed from a codon-optimized gene – that is produced at roughly 100-fold lower levels. This bodes well for possible large-scale application of CmINV and could provide an alternative to other invertases being used in industry.

To our knowledge, no other gene product from *C. moniliformis* has been characterized to any extent. Although the complete genome sequence of *C. moniliformis* CMW 10134 is not currently publicly available, this work highlights how gene discovery is facilitated in the exciting era of next-generation sequencing and the vast amounts of genomic data associated with it. By making use of reverse genetics approaches such as the one used in this study, a plethora of industrially functional products await discovery, not only in *Ceratocystis* but all of the thousands of fungi whose genomes are currently being sequenced (e.g. http://1000.fungalgenomes.org).

## Conclusions

We described here the β-fructofuranosidase gene (*CmINV*) from *C. moniliformis* and the recombinant expression thereof in *S. cerevisiae*. The recombinant CmINV showed high affinity towards sucrose and along with its notable high-level production and secretion by *S. cerevisiae* makes this protein an attractive option for large-scale applications.

## Methods

### Strains, culture conditions and DNA manipulations

General procedures for cloning, DNA isolation, amplification and manipulations, transformations, agarose and protein gel electrophoresis were performed as described by Sambrook et al. [32]. *Escherichia coli* DH5α served as a cloning host and for plasmid propagation and was routinely cultivated at 37°C in LB media (10 g/L tryptone, 10 g/L NaCl, 5 g/L yeast extract) supplemented with 100 μg/mL ampicillin. The *C. moniliformis* genome sequence was derived from strain CMW 10134 (Fungal and Yeast Collection, Centraalbureau voor Schimmelcultures, WDCM no.: WDCM133, http://www.cbs.knaw.nl/) and used to identify and clone the *CmINV* gene. *C. moniliformis* CMW 10134, originally isolated from *Eucalyptus grandis* trees in South Africa [33], was cultured and maintained on Potato Dextrose Agar (PDA) (BD Difco). The *SUC2* deletion strain of *S. cerevisiae* BY4742 (Mat α; *his3*∆*1*; *leu2*∆*0*; *lys2*∆*0*; *ura3*∆*0*; *YIL162w*::*kanMX4*) (EUROSCARF collection) [33,34] was used for the heterologous expression of the *CmINV* gene. Transformed *S. cerevisiae* BY4742 cells were cultivated aerobically at 30°C shaken at 200 rpm in synthetic complete (SC) media containing 6.7 g/L yeast nitrogen base, 1.3 g/L amino acid pool without uracil and 20 g/L glucose (referred to SC_Gluc_ from here on) or sucrose (referred to SC_Suc_ from here on). SC media was also buffered with 20 g/L succinic acid, pH adjusted to 6.0 with 10 N NaOH and with/without 1.5% (w/v) agar.

### CmINV gene identification and construction of *S. cerevisiae* expression vectors

The genome sequence of *C. moniliformis* CMW 10134 was sequenced using 454 pyrosequencing technology at Inqaba Biotechnology (Roche Diagnostics). Sequence reads were assembled into 600 contigs using Newbler version 2.3 then subjected to local BLAST searches to identify the contig encoding the *CmINV* gene. This was done using the *Aspergillus niger* (recently renamed *Aspergillus japonicus*[15]) ATCC 20611 *fopA* amino acid sequence (GenBank accession no. BAB67771) and BioEdit (http://www.mbio.ncsu.edu/BioEdit/bioedit.html). By making use of the online annotation programme AUGUSTUS [35] one open reading frame of 1848 bp was identified within this contig as having β-fructofuranosidase-like domains. The predicted amino acid sequence for *CmINV* was compared to the NCBI database using BLASTp, while SignalP 4.0 (http://www.cbs.dtu.dk/services/SignalP/) and NetNGlyco 1.0 (http://www.cbs.dtu.dk/services/NetNGlyc/) were used to analyse the relevant features of the predicted protein sequence.

Genomic DNA extracted from fresh *C. moniliformis* CMW 10134 mycelia grown on PDA was used to amplify the *CmINV* gene [36]. The primers CmINV-F (5’-ACTG*TTAATTAA* ***GAATTC*** **ATG**GTTTTCCAATCTTCTGC-3`) and CmINV-R (5’-ACTG ***GAATTC*** *GGCGCGCC***TTA**CTCAATGTAAGTCAGAG-3`) were designed to amplify the *CmINV* coding region (start and stop codons in bold) based on the *CmINV* genomic sequence to include targets for the *Eco*RI restriction enzyme (bold italicised) as well as auxiliary targets for *Pac*I (in CmINV-F) and *Asc*I (in CmINV-R) restriction enzymes (italicised). PCR amplification was conducted using the Phusion High-Fidelity PCR polymerase (Thermo Scientific) according to the manufacturer’s instructions in a MultiGene™ Gradient PCR Thermal Cycler (Labnet). The 1850-bp amplicon was digested with *Eco*RI and ligated with T4 ligase (Thermo Scientific) into an *Eco*RI-digested *E. coli*/*S. cerevisiae* shuttle vector pJC1 (containing the constitutive *S. cerevisiae* phosphoglycerate kinase 1 (*PGK1*) promoter and terminator cassette) [37].

The *fopA* gene encoding an *Aspergillus niger* ATCC 20611 β-fructofuranosidase (GenBank accession no. AB046383) was synthesized by GeneArt (Regensburg, Germany) and used as a positive control during β-fructofuranosidase expression studies in *S. cerevisiae*. The *fopA* native secretion signal was replaced by the *Trichoderma reesei* endo-β-1,4-xylanase 2 (*Xyn11A*) secretion signal. This synthetic construct was subcloned as a 2033-bp *Eco*RI-*Xho*I fragment from the in-house pMK-RQ cloning vector to the *Eco*RI-*Xho*I digested pJC1 vector.

The pJC1-*CmINV*, pJC1-*fopA* plasmids and the empty pJC1 vector were transformed into the *S. cerevisiae* BY4742 strain using a lithium acetate-DMSO method [38]. *S. cerevisiae* BY4742[CmINV], BY4742[fopA] and BY4742[pJC1] transformants were confirmed with PCR. As an indication of β-fructofuranosidase functionality, the (in)ability of the *S. cerevisiae* BY4742[CmINV], BY4742[fopA] and BY4742[pJC1] yeast transformants to grow on sucrose as sole carbohydrate source was performed on SC_Suc_.

### Purification of CmINV

The *S. cerevisiae* BY4742[CmINV] and BY4742[fopA] strains were grown in above mentioned SC_Gluc_ media for three days. Two millilitres of supernatant were applied onto a Superose 12 HR 10/30 (GE Healthcare Bio-Sciences) column equilibrated in phosphate-buffered saline buffer pH 6.5. The column was coupled to an Äkta FPLC purifier (Amersham Biosciences) and Unicorn version 4.00 software was used to process data. With a flow rate of 1 ml/min, fractions of 500 μL were taken after which samples were assayed for invertase activity and analysed on polyacrylamide gels. Further characterization was done on fractions that appeared electrophoretically homogenous after silver staining. For the determination of protein concentration of fractions, the Lowry-based DC protein assay kit (BioRad) was used. Bovine serum albumin at a concentration range of 0.247-1.48 mg/mL was used to construct a standard curve.

### Enzyme assays

Enzyme assays were based on reducing sugars released from the cleavage of sucrose to fructose and glucose using the 3,5-dinitrosalicyclic acid (DNS) [39]. Assays were conducted in either triplicate or quadruplicate using a MultiGene™ Gradient PCR Thermal Cycler (Labnet). In short, five microliters of appropriately diluted enzyme were added to 45 μL of 50 mM sucrose substrate and incubated at predetermined temperatures in the PCR thermocycler. The reaction was terminated after five minutes of incubation with the addition of a solution containing 1% DNS, 20% potassium sodium tartrate, 1% NaOH, 0.2% phenol, and 0.05% Na_2_SO_3_. The samples were then boiled for five minutes and the colour reaction recorded spectrophotometrically at 540 nm. International units (IU) of enzyme were defined as the amount of product (in μmole) released per minute under experimental conditions. A standard curve was constructed using glucose concentrations of 0.625-10 mM from which eventual IU values were calculated.

Optimum pH and temperature conditions were determined using assays that were all conducted with 50 mM sucrose in 50 mM citrate-phosphate buffers of different pH (3.0-7.0) and at different temperatures (35-69°C). For the thermostability test, the enzyme samples were pre-incubated at different temperatures (35-60°C, in 5°C increments) at pH 6.0 for a set period of time after which similar DNS-based assays were conducted as described earlier. For the determination of kinetic parameters, sucrose concentrations that ranged from 3 mM to 50 mM were used. *K*_m_ and *V*_max_ estimations were determined by nonlinear regression analysis of velocity versus substrate concentration plots, using the Solver function of Microsoft Excel [40]. To determine the influence of different metal ions on invertase activity, assays were conducted using purified enzyme with two different concentrations of metal solution (5 and 50 mM) and were compared to the control i.e. with no metal ions present in the sucrose solution. Assays for recombinant fopA were conducted similarly as described above, but at its optimal conditions of 68°C at pH 5.0.

Enzyme assays destined for scFOS analyses for both CmINV and fopA were conducted at 40°C in 50 mM citrate-phosphate buffer pH 6.0. Five-hundred microliters of crude extract of *S. cerevisiae* BY4742[CmINV] and BY4742[fopA] (appropriately diluted to equate to one IU of enzyme) were added to 500 μL of 10% v/v (0.29 M) sucrose and incubated for 60 minutes. The assay reaction was terminated by adding perchloric acid up to 2% (v/v). After adding 48 μL of 7 N KOH, samples were filtered through a 0.22 μm syringe filter and were analysed for scFOS production. The fructosyltransferase activity (U_t_) and hydrolysis activity (U_h_) were measured from the amounts of scFOS and glucose, respectively.

### High-performance anion exchange chromatography with pulsed amperometric detection (HPAEC-PAD)

Samples (10 μl) were analysed on a Dionex Ultimate 3000 system equipped with a Coulochem III electrochemical detector with working gold electrode operating in the pulsed amperometric mode. HPLC-grade standards for L-arabinose, D-glucose, D-fructose and D-sucrose were purchased from Sigma-Aldrich. L-arabinose served as an internal standard. The fructooligosaccharide set (1-kestose, 1-nystose and 1^F^-fructofuranosylnystose) was purchased from Wako Chemicals GmbH. Mobile phases were made using sodium acetate (Sigma-Aldrich, cat. no.: 71180) and 50% NaOH solution (Fluka, cat. no.: 71686) according to recommendations by Dionex. Ultra-pure 18 MΩ deionized water used in experiments was obtained from a Milli-Q UF Plus system (Millipore). Separation of sugars was achieved by gradient elution on a CarboPac PA1 (4 × 250 mm) analytical column coupled to a PA1 (4 × 50 mm) guard column. A LPG-3400AB pump generated the required gradients by mixing solvents A (250 mM NaOH), B (100 mM NaOH with 500 mM sodium acetate) and C (water) according to the program described in Additional file 2: Table S1. The flow rate was set at 1 ml/min. The PAD settings were for a quadruple-potential waveform [41] with a minor modification of the E3 pulse set at 600 mV for 10 ms. The assembly was controlled by a desktop computer running the Chromeleon 6.8 Chromatography Data System software. Sugars were identified and quantified by comparing the retention times and ratios of sample peak area to internal standard peak area to similar ratios determined for external standards.

### Electrophoresis and zymogram analyses

Crude extract and purified protein samples were subjected to 10% SDS-PAGE. Loading dye consisted of 60 mM Tris-HCl (pH 6.8), 25% glycerol, 2% SDS, 14 mM β-mercaptoethanol and bromophenol blue and gels were run in Tris-glycine buffer (25 mM Tris-HCl, 250 mM glycine, 0.1% SDS). After electrophoresis, protein bands were visualized with a silver-stain procedure [42]. For zymogram analyses, after electrophoresis, gels were washed in 50 mM citrate-phosphate buffer pH 6.0 for 60 minutes to remove SDS. Gels were then incubated at 60°C in the presence of 50 mM sucrose for 15 minutes. After removal of sucrose solution, gels were placed in a boiling 100 mM NaOH solution that contains 0.2% triphenyltetrazolium chloride (Sigma) [43]. Some protein samples were de-*N*-glycosylated overnight with the endoglycosidase PNGase F (New England Biolabs) according to the manufacturer's protocol, prior to SDS PAGE.

### Phylogenetic analyses

The relationship between the CmINV and other known invertases was determined by making use of a previous phylogenetic analysis of the GH32 family [15]. The *Ceratocystis CmINV* sequence was manually added to an alignment for this protein family, which were obtained from Timothy Y. James (University of Michigan, Ann Arbor, MI, USA). The dataset was then subjected to phylogenetic analyses using maximum likelihood and Bayesian Inference approaches based on the models and parameters previously used [15].

### Nucleotide sequence accession number

The nucleotide sequence reported here for *CmINV* has been deposited in GenBank database under accession number KF129393.

## Abbreviations

BLAST: Basic local alignment sequencing tool; bp: Base pair; DC: Detergent compatible; DNS: Dinitrosalicylic acid; EC: Enzyme class; FPLC: Fast protein liquid chromatography; GF: Sucrose; GF2: 1-Kestose; GF3: 1-Nystose; GF4: 1^F^-Fructofuranosylnystose; GH: Glycoside hydrolase; HPAEC: High-performance anion exchange chromatography; HPLC: High-performance liquid chromatography; IU: International units; Km: Michaelis constant; LB: Luria Bertani; MW: Molecular weight; PAD: Pulsed amperometric detection; PDA: Potato dextrose agar; SC: Synthetic complete; scFOS: Short-chain fructooligosaccharides; Uh: Hydrolase activity; Ut: Transferase activity; Vmax: Maximal reaction velocity.

## Competing interests

The authors declare that they have no competing interests.

## Authors’ contributions

The NVW and KMT conducted all cloning experiments. NVW purified and characterized the CmINV protein. KMT did all the HPAEC analyses. KMT, BDW, ETS, HV conducted all the bioinformatic work including the phylogeny. NVW drafted the manuscript. All authors contributed in revising the initial draft. All authors read and approved the final version.

## Supplementary Material

Additional file 1 **(a) Superose size-exclusion chromatography profile of *S. cerevisiae* BY4742[CmINV] supernatant containing CmINV with invertase activity of each fraction. (b)** silver-stained SDS-PAGE gel of pooled fractions (fraction 4 and 5). Click here for file

Additional file 2 **HPAEC-PAD method for the gradient for elution of sugars.** Gradient mixing program for high-performance anion exchange chromatography (HPAEC-PAD). Click here for file
