# Supplementary figures and images for: Identification of the gene for β-fructofuranosidase from Ceratocystis moniliformis CMW 10134 and characterization of the enzyme expressed in Saccharomyces cerevisiae
Source: BMC Biotechnol. 2013 Nov 14;13:100. doi: 10.1186/1472-6750-13-100 (PMC3880211; doi:10.1186/1472-6750-13-100)

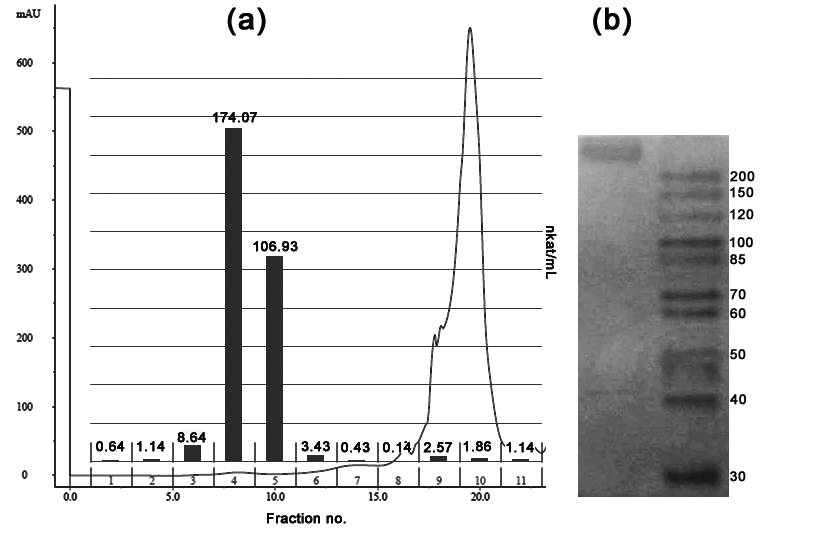

Supplement: Additional file 1 — (a) Superose size-exclusion chromatography profile of S. cerevisiae BY4742[CmINV] supernatant containing CmINV with invertase activity of each fraction. (b) silver-stained SDS-PAGE gel of pooled fractions (fraction 4 and 5). [file 1472-6750-13-100-S1.tiff]
