# Supplementary material for: Identification of the gene for β-fructofuranosidase from Ceratocystis moniliformis CMW 10134 and characterization of the enzyme expressed in Saccharomyces cerevisiae
Source: BMC Biotechnol. 2013 Nov 14;13:100. doi: 10.1186/1472-6750-13-100 (PMC3880211; doi:10.1186/1472-6750-13-100)
Supplement: Additional file 2 — HPAEC-PAD method for the gradient for elution of sugars. Gradient mixing program for high-performance anion exchange chromatography (HPAEC-PAD). [file 1472-6750-13-100-S2.docx]

Supplementary Table 1. HPAEC-PAD method for the gradient for elution of sugars.

| **Time** | **% A** | **% B** | **% C** |
| --- | --- | --- | --- |
| 0 | 9 | 0 | 91 |
| 10 | 52 | 0 | 48 |
| 16 | 69 | 21 | 10 |
| 23 | 69 | 21 | 10 |
| 23.2 | 9 | 0 | 91 |
| 40.0 | 9 | 0 | 91 |

A: 250 mM sodium hydroxide; B: 100 mM NaOH with 500 mM sodium acetate; C: Water. Flow rate was 1 ml/min.
